# Concepts of Ethics and Their Application to AI

**DOI:** 10.1007/978-3-030-69978-9_3

**Published:** 2021-03-18

**Authors:** Bernd Carsten Stahl

**Affiliations:** Centre for Computing and Social Responsibility, De Montfort University, Leicester, UK

**Keywords:** Ethical theory, Human flourishing, Purposes of AI, Ethical principles for AI

## Abstract

Any discussion of the ethics of AI needs to be based on a sound understanding of the concept of ethics. This chapter therefore provides a brief overview of some of the key approaches to ethics with a particular emphasis on virtue ethics and the idea of human flourishing. The chapter reviews the purposes for which AI can be used, as these have a bearing on an ethical evaluation. Three main purposes are distinguished: AI for efficiency, optimisation and profit maximisation, AI for social control and AI for human flourishing. Given the focus on human flourishing in this book, several theoretical positions are introduced that provide insights into different aspects and ways of promoting human flourishing. The chapter concludes with a discussion of the currently widespread principle-based approach to AI ethics.

Ethical issues of AI are hotly debated and sometimes contested. In order to understand what they are and why they might be considered ethical issues, and to start thinking about what can or should be done about them, I start with an introduction to ethics, which is then followed by an empirically based discussion of current ethical issues of AI.

At its most basic level, ethics has to do with good and bad, with right and wrong. However, the term “ ethics” is much more complex than that and the same word is used to cover very different aspects of the question of right and wrong. Elsewhere (Stahl 2012), I have proposed the distinction of four different levels, all of which are covered by the term “ ethics”:

1. Moral intuition, expressed in a statement of the sort: “This is right,” or “This is wrong.”
2. Explicit morality, expressed in general statements like “One should always /never do this.”
3. Ethical theory, i.e. the justification of morality drawing on moral philosophy expressed in statements like “Doing this is right/wrong because …”
4. Metaethics, i.e. higher-level theorising about ethical theories.

This view of ethics is compatible with other views, notably the frequently suggested distinction between applied ethics, normative ethics and metaethics. It also accommodates the typical introduction to ethics that one can find in technology ethics textbooks (Van de Poel and Royakkers 2011), notably the dominant ethical theories of deontology and consequentialism.

## Ethical Theories

Ethical theories are attempts to find an answer to the question: what makes an action ethically better or worse than an alternative action? Prominent examples of ethical theories include consequentialism and deontology. (I shall return to virtue ethics later.) Both of these originated during the Enlightenment period (mainly in the 18th century). They aim to provide clear rules that allow us to determine the ethical quality of an action. Consequentialist theories focus on the *outcomes* of the action for this evaluation. The various approaches to utilitarianism going back to Jeremy Bentham (1789) and John Stuart Mill (1861) are the most prominent examples. They are based on the idea that one can, at least in theory, add up the aggregate utility and disutility resulting from a particular course of action. The option with the highest net utility, i.e. utility minus disutility, is the ethically optimal one.

Deontology, on the other hand, is based on the principle that the basis of the ethical evaluation of an action is the duty of the agent executing it. The most prominent representative of this position is Immanuel Kant (1788, 1797), who formulated the so-called categorical imperative. The most often quoted formulation of the categorical imperative says “Act only on that maxim by which you can at the same time will that it should become a universal law” (translation, quoted in Bowie 1999: 14). This categorical imperative stops agents from rationalising exemptions for themselves. The interesting aspect of such a position for our purposes is that this view of ethics pays no immediate attention to the consequences of an action, but exclusively focuses on the motivation for undertaking it.

It is important to underline, however, that deontology and utilitarianism are not the only ethical theories that can be applied to AI, and to technology more broadly. In addition to virtue ethics, to which I will return shortly, there are other general ethical approaches such as the feminist ethics of care (Gilligan 1990) and ethics based on various religions. Applying ethical theories to particular application areas has resulted in rich discourses of concepts such as computer ethics (Bynum and Rogerson 2003, Bynum 2008a, van den Hoven 2010), information ethics (Capurro 2006, Floridi 2006) and technology ethics (Brey 2011) that are relevant to AI.

Entire libraries have been written about philosophical ethics, and I cannot hope to do justice to the many and rich nuances of ethical thinking. It may nevertheless be helpful to outline how ethics links to the human condition. This can explain some of the characteristics of ethics and it can shed light on whether or to what degree non-human artificial agents can be ethical subjects.

A key to understanding ethics, I believe, is that humans recognise that we all, despite many and far-reaching differences, have much in common. We could call this state “the shared features of the human condition”. Human beings are fundamentally social. Without social structures and support we would not only die as infants, but also fail to develop the language and thus the conceptual understanding of the world around us that allow us to live our lives. We are possibly the only species that not only recognises that we exist but also knows that we are fundamentally vulnerable and mortal. We not only know this, but we feel it in profound ways, and we recognise that we share these feelings with other humans. The shared fate of certain death allows us to see the other as someone who, no matter how different from us they are, has some basic commonalities with us. We have empathy with others based on our experiences and the assumptions that they are like us. And just as we share the knowledge of death, we also share the experience of hope, of joy, of the ability to (more or less) freely develop projects and shape our world. This world is not just a physical world, but predominantly a social one, which is constructed using the unique capabilities of human language. Ethics is then a way to shape an important part of this social world in ways that take into account the shared aspects of human nature.

This description of human nature and the human condition has direct implications for the concept of ethics and what can count as “being ethical”. Ethics does not exclusively reside in an action or an intention. Ethics is part of *being* in the world, to use a Heideggerian term (Heidegger 1993). It is characterised by an agent’s ability not only to perceive different possible states of the world and decide between conceivable options, but to do so with a view to the meaning of such a decision for her own world and also for the world at large. This implies that the agent is consciously situated in this world, and understands it, but also has an emotional relationship to it and the fellow agents who co-constitute this world. Such an agent may very well make use of deontological or utilitarian ethical theories, but she does so in a reflective way as an agent who has a commitment to the world where these principles are applied.

This brief introduction to my ethical position points to the idea of human flourishing, which will become vital in later parts of this book: human flourishing linked to *being* in the world, understanding the limits of the human condition and the essential socialness of humans, which requires empathy. Of course, I realise that there are people who have no or little empathy, that abilities to interact socially and use language differ greatly, that many of these aspects apply to some degree also to some animals. Yet, to substantiate my position in AI ethics and the main ideas of this book, it is important that I do not draw inordinately on deontology and utilitarianism, but rather take into account a wider range of sources, and in particular virtue ethics.

## AI for Human Flourishing

Current approaches to philosophical ethics as represented by consequentialism and deontology are largely rational and theoretical endeavours and mostly at home in academic philosophy departments. Ethics, however, has traditionally had a much broader meaning. For the ancient Greeks, philosophy was not just an intellectual endeavour but an attempt to find ways to live the “good life”, the answer to the question: how should I live (Annas 1993)? The major philosophical schools of ancient Greece agreed that the cosmos had a purpose and that the individual good life, resulting in happiness (Aristotle 2007), was predicated on people fulfilling their role in society. This is the basis of virtue ethics, which is most prominently associated with Aristotle (2007) but whose main tenets are widely shared across philosophical schools. The focus of this approach to ethics is not so much the evaluation of the anticipated outcomes of an individual act or their intention, but providing guidance for the individual to help them develop a virtuous character.

I do not want to overly romanticise ancient Greece, whose acceptance of slavery and misogyny are not acceptable. However, virtue ethics as an approach to ethics has significant appeal, probably because it offers to provide guidance not only on individual problems but on how we should live our lives. This may explain why it has returned to prominence since the end of the 20th century and seen attempts to translate it into modern contexts (MacIntyre 2007).

Terry Bynum is one of several scholars who have succeeded in translating the ancient principles of virtue ethics into a modern technology-saturated context. He suggests the development of a “flourishing ethics” (Bynum 2006) which draws from Aristotelian roots. Its key tenets are:

1. Human flourishing is central to ethics.
2. Humans as social animals can only flourish in society.
3. Flourishing requires humans to do what we are especially equipped to do.
4. We need to acquire genuine knowledge via theoretical reasoning and then act autonomously and justly via practical reasoning in order to flourish.
5. The key to excellent practical reasoning and hence to being ethical is the ability to deliberate about one’s goals and choose a wise course of action.

Bynum (2008b) has shown that these principles of virtue ethics are relevant to and have informed ethical considerations of information technology since its early days and can be found in the work of Norbert Wiener (1954), one of the fathers of digital technology.

Much research has been undertaken to explore how principles of virtue ethics can be applied to technology and how we can live a virtuous life in a technologically driven society. An outstanding discussion of virtue ethics in the context of digital technologies is provided by Vallor (2016), and, given that my approach relies heavily on her discussion, I will return to it later with reference to human flourishing.

As Bynum points out, people are endowed with different skills and strengths. Flourishing includes excellence in pursuit of one’s goals, which implies that there are as many ways of flourishing as there are combinations of skills. Flourishing is thus not a one-size-fits-all concept but needs to be filled with life on an individual level. Before I return to a more detailed discussion of the concept of flourishing, I now want to discuss the motivations behind and purposes of developing, deploying and using AI, as these have a direct bearing on the ethical evaluation of AI socio-technical systems.

## Purposes of AI

**Fig. 3.1:**
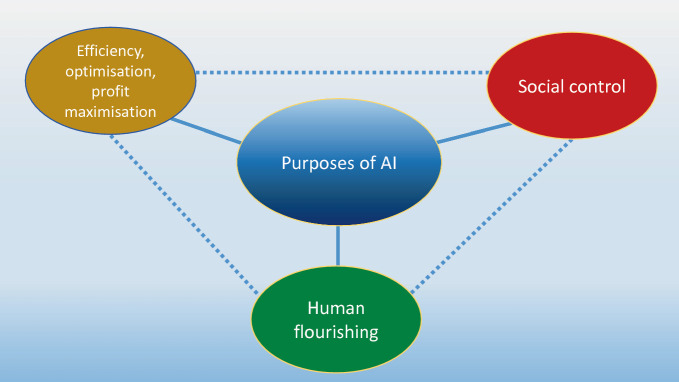
Possible purposes of AI

Understanding the purpose and intention of AI is important when thinking about the ethics of AI. Digital technologies, as pointed out earlier, are highly flexible and open to interpretation. They are logically malleable. They can thus be used for an infinity of purposes, which may or may not be aligned with the intention of the original developers and designers. Despite this openness of AI, it is still possible to distinguish different purposes that determine the design, development and use of systems. I distinguish three main purposes: AI for efficiency, AI for social control and lastly, as an alternative and complement to the two initial ones, AI for human flourishing (see Fig. 3.1).

When looking at current policy documents covering AI, one typically finds a mixture of all three of these motivations: AI can *improve* *efficiency*, which will lead to cost savings and thereby to economic benefits, which will trickle down, and people’s lives will get better. A report to the President of the United States set the tone by highlighting the economic advantages and suggesting that “AI has the potential to double annual economic growth rates in the countries analyzed by 2035” (Executive Office of the President 2016). The European Commission expects that “AI could spread across many jobs and industrial sectors, boosting productivity, and yielding strong positive growth” (Craglia et al. 2018). And a committee of the United Kingdom’s House of Lords hopes that “AI could spread across many jobs and industrial sectors, boosting productivity, and yielding strong positive growth” (House of Lords 2018).

A very different view of the use of technology including AI is to see it as a way of exerting *social control*. Rapidly growing abilities to collect data, in conjunction with AI’s ability to detect patterns and correlations between variables, allow for new ways of controlling human behaviour. This can be done in subtle ways, using the idea of “nudging” based on behavioural economics (Mullainathan and Thaler 2000, Camerer et al. 2004) or it can be done more vigorously, as for example in the Chinese social credit scoring system (Creemers 2018, Liu 2019).

The system intends to monitor, rate and regulate the financial, social, moral and, possibly, political behavior of China’s citizens – and also the country’s companies – via a system of punishments and rewards. The stated aim is to “provide the trustworthy with benefits and discipline the untrustworthy.” (Bartsch and Gottske nd)

AI as social control can also breach the limits of legality, as happened in the Facebook–Cambridge Analytica case, where social media data was used to illegitimately influence the outcome of democratic elections (Isaak and Hanna 2018). Zuboff (2019) offers a forceful argument that social control is a driving force and a necessary condition of success of what she calls “ surveillance capitalism”. In her analysis she does not focus on the term AI, but her description of the way in which new business models have developed and facilitated enormous profits is fully aligned with the concept of AI as converging socio-technical systems (see Fig. 3.1).

The third purpose of using AI, drawing on the earlier discussion of ethics, is to employ it for *human flourishing*. This means that AI is developed and deployed in ways that promote human flourishing. It can be used as a tool that helps individuals and groups identify worthwhile goals and supports them in their pursuit of excellence in achieving these goals. There are a number of suggestions on how to ensure that AI has positive consequences for individuals and societies, which is part of this third purpose of using AI for human flourishing: for example, attempts to construct a “good AI society” (Cath et al. 2016) or the discourse on AI for good that I discuss in more detail below in the section on the benefits of AI.

The three different views of the purpose of AI are represented in Fig. 3.1.

These three goals may come into conflict, but they are not necessarily contradictory.

The pursuit of efficiency and the resulting economic benefits can lead to a strong economy that provides the material substrate for human wellbeing. By generating wealth an efficient economy opens avenues of human flourishing that would otherwise be impossible. For instance, a move from coal-based energy production to solar energy is expensive. In addition, the pursuit of efficiency and profit creation can be a legitimate area of activity for excellence, and people can flourish in this activity.

Social control is often seen as problematic and in conflict with individual liberties. The use of information and communications technologies (ICTs) has long been associated with violations of privacy and the growth of surveillance (Lyon 2001). This concern traditionally saw the state as the source of surveillance. In these days of corporate giants that control much of the data and technical infrastructure required for AI, the concern includes the exploitation of individuals in new forms of “ surveillance capitalism” (Zuboff 2019). But, again, there does not have to be a contradiction between social control and human flourishing. Humans as social beings need to define ways of collaborating, which includes agreement on moral codes, and these need to be controlled and enforced in some way. While nudging as a policy instrument is contentious, it can be and often is used to promote behaviours that are conducive to flourishing, such as promoting a healthier lifestyle. Used especially in the United Kingdom, Australia, Germany and the US (Benartzi et al. 2017), nudging involves government-led campaigns to achieve given targets, for instance higher vaccination rates. For example, a US campaign involved sending out planning prompts for flu vaccination to citizens, which increased vaccination rates by 4.2% (ibid).

**Fig. 3.2:**
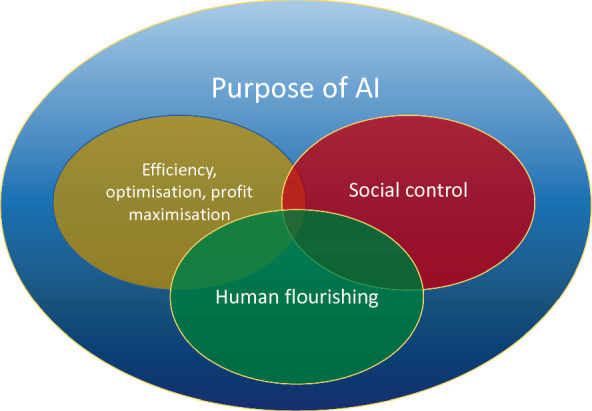
Overlap of purposes of AI

In the technology domain AI can be used to promote privacy awareness (Acquisti 2009), arguably a condition of flourishing. As I write these sentences, much of the world is under lockdown due to the COVID-19 pandemic. In the UK there is a heated debate around apps to be used to support the tracking and tracing of infected individuals (Klar and Lanzerath 2020). What this shows is that even forced social control through digital technologies may in some circumstances be conducive to human flourishing, for example, if it can help save lives and allow society to function. A Venn-type diagram may therefore be a better representation of the relationship of the three purposes (Fig. 3.2).

I must emphasise that the three purposes of AI listed in Figures 3.1 and 3.2 are not intrinsically contradictory, but rather describe the main fields of emphasis or different directions of travel that can guide the development and deployment of AI. My proposal is that the explicit aim to do the ethically right thing with AI can be described with reference to human flourishing.

This is not a novel insight. It draws from the ancient Greek philosophers and has been applied to ICT for decades. It has also been applied to AI. Virginia Dignum (2019: 119), for example, states: “Responsible Artificial Intelligence is about human responsibility for the development of intelligent systems along fundamental human principles and values, to ensure human flourishing and well-being in a sustainable world.” Mark Coeckelbergh (2019: 33) voices a similar view when he states that we “need a positive and constructive ethics of AI, which is not only about regulation in the sense of constraints but which also concerns the question of the good life and human and societal flourishing”. The principle of this argument is unproblematic and can also be found in AI policy proposals (ALLEA and Royal Society 2019). Who, after all, would say that they want to use AI to limit human flourishing? However, it raises the questions: how can we know whether human flourishing is promoted or achieved, and how can this be translated into practice? In order to answer these questions, I will now look at some theoretical positions on technology and its role in the world.

## Theoretical Perspectives on Human Flourishing

Flourishing ethics is part of the tradition of virtue ethics and its historical roots in Aristotelian ethics. In order to answer the question, “How can we understand flourishing in practical terms?” it is helpful to look at other positions that share the aim of promoting human flourishing. Three positions that have been applied to technology, or that were developed specifically with research and technology development in mind, are important in this context: critical theory of technology, capability theory and responsible research and innovation. Each of these three offers an established theoretical approach that is consistent with human flourishing, and each has led to a wealth of insights into how flourishing can be observed and promoted..

*Critical theory* *of technology* is my first example of a theoretical approach relevant to AI that encompasses flourishing. Critical theory has a number of different possible roots. In its European spirit it tends to trace its origins to Marx’s criticism of capitalism. There is a recurrence of Marxist thinking in relation to digital technologies (Greenhill and Wilson 2006, Fuchs and Mosco 2017). However, much of critical theory of technology uses later developments of critical theory, notably of the Frankfurt School (Wiggershaus 1995). Andrew Feenberg’s (1993, 1999) work is probably the best-known example of the use of critical theory to understand modern technology. In addition, there has been a long-standing discussion of critical theory in the field of information systems, which draws on further theoretical traditions, such as postcolonialism (Mayasandra et al. 2006) and postmodernism (Calás and Smircich 1999).

Elsewhere I have argued that one central combining feature of the various different views of critical theory is that they aim to promote emancipation (Stahl 2008). The emancipatory intention of critical research, i.e. research undertaken in the critical tradition, means that resulting research cannot be confined to description only, but attempts to intervene and practically promote emancipation (Cecez-Kecmanovic 2011). Myers and Klein (2011), drawing on Alvesson and Willmott (1992), see emancipation as facilitating the realisation of human needs and potential, critical self-reflection and associated self-transformation. The concept of emancipation seems very close to the principle of human flourishing discussed earlier. My reason for bringing critical theory into this discussion is that critical theory has developed a set of tools and a high degree of sensitivity for understanding factors that can impede emancipation. Because of its roots in Marxist ideology critique, critical theory is well positioned to point to the factors limiting emancipation and flourishing that arise from the current socio-economic system, from labour processes and from capitalist modes of production. As will be seen later, these constitute probably the largest set of ethical issues associated with AI.

A second theoretical position worth highlighting in the context of human flourishing is *capability theory*. Capability theory has roots in philosophy and economics and is strongly associated with Amartya Sen (2009) and Martha Nussbaum (2011). The capability approach originated in development economics and the desire to find better ways of describing human development than purely financial and aggregate measures such as the gross domestic product. It is also directly linked to and based on the Aristotelian notion of flourishing (Johnstone 2007), and thus immediately relevant to a discussion of the ethics of AI and human flourishing.

The reason for highlighting the capability approach is that it has a history of application to information technologies (Oosterlaken and van den Hoven 2012), often in the context of studies of ICT for development and its focus on marginalised and vulnerable populations (Kleine 2010). It can thus be used as a way of sharpening the focus on the impact that AI can have on such populations. In addition, the communities working with the capability approach have developed tools for improving human functioning and freedoms and for measuring outcomes that have been recognised at a political level, notably by the United Nations. It is therefore suited to the creation of metrics that can be used to assess whether AI applications and uses benefit human flourishing.

The final theoretical position relevant to AI ethics and human flourishing is that of *responsible research and innovation* (RRI). RRI is a concept that has gained prominence in research and innovation governance since around the early 2010s. It has been defined as the “on-going process of aligning research and innovation to the values, needs and expectations of society” (European Union 2014). There are different interpretations of RRI (Owen and Pansera 2019), including that of the European Commission (2013), which consists of six pillars or keys (engagement, gender equality, science education, ethics, open access and governance), and that of the UK’s Engineering and Physical Sciences Research Council (Owen 2014), represented by the AREA acronym (anticipate, reflect, engage and act), which is based on Stilgoe et al. (2013).

A much-cited definition of RRI proposed by Von Schomberg (2013: 63) sees RRI as

a transparent, interactive process by which societal actors and innovators become mutually responsive to each other with a view to the (ethical) acceptability, sustainability and societal desirability of the innovation process and its marketable products (in order to allow a proper embedding of scientific and technological advances in our society).

The reference to RRI is helpful in the context of AI ethics because it puts research and innovation explicitly into the societal context. The idea that the process and product of research and innovation should be acceptable, sustainable and societally desirable can be read as implying that they should be conducive to human flourishing. RRI can thus be understood as a way of promoting and implementing human flourishing. RRI is important in the context of this book because it is established as a term in research funding and familiar to policymakers. A recent proposal by the European Parliament puts heavy emphasis on RRI as a way to ensure ethical sensitivity in future AI research, development and deployment. The European Parliament (2020: 6) suggests that “the potential of artificial intelligence, robotics and related technologies … should be maximized and explored through responsible research and innovation”.

Human flourishing in the broad sense used here is something that I believe most people can sign up to. It does not commit us to a particular way of life or require the adoption of a particular ethical position. It does not prevent us from using other ethical theories, including deontology and utilitarianism, to assess ethical questions (Bynum 2006). It is compatible with various theoretical positions beyond the three ( critical theory, capability theory, RRI) introduced here. The choice of human flourishing was guided by the need to find an ethical language that can find traction across disciplinary, national, cultural and other boundaries. AI technologies are global and pervasive, but they have an impact at the local and individual level. An approach to the ethics of AI that aims to provide general guidance therefore needs to be able to build bridges across these many global divides, which I hope the idea of flourishing does.

## Ethical Principles of AI

The main thesis of this book is that flourishing ethics can enlighten AI ethics and provide guidance in the development of practical interventions. The majority of currently existing guidelines were not drafted from one theoretical viewpoint but tend to use a set of ethical principles or values. What are these values?

The most comprehensive review of AI ethics guidelines published so far (Jobin et al. 2019) lists the following ethical principles: transparency, justice and fairness, non-maleficence, responsibility, privacy, beneficence, freedom and autonomy, trust, sustainability, dignity and solidarity. Each of these is comprised of components. Transparency, for example, refers to related concepts such as explainability, explicability, understandability, interpretability, communication and disclosure. The relationship between these concepts is not normally well defined and they can refer to different ethical positions. Elsewhere we have tried to clarify their normative implications (Ryan and Stahl 2020).

Another example, the ethics guidelines for trustworthy AI proposed by the EU’s High-Level Expert Group on Artificial Intelligence (2019), has a tiered level of principles. The expert group proposes a framework for trustworthy AI that consists of lawful AI (which they do not cover), ethical AI and robust AI. This framework is based on four ethical principles: respect for human autonomy, prevention of harm, fairness and explicability. From these principles they deduce seven key requirements for the realisation of trustworthy AI, namely:

1. human agency and oversight
2. technical robustness and safety
3. privacy and data governance
4. transparency
5. diversity, non-discrimination and fairness
6. social and environmental wellbeing
7. accountability.

From these they then develop assessment methods for trustworthy AI and policy recommendations.

It is easy to see the attraction of this principle-based approach. It avoids making strong commitments to typically contested ethical theories. The principles themselves are generally uncontroversial, thereby offering the opportunity of a consensus. Maybe most importantly, the principle-based approach has been the basis of biomedical ethics, the field of ethics with the longest history of high-visibility public debate and need for societal and political intervention. Biomedical ethics in its modern form resulted from the Nazi atrocities committed during research on humans in concentration camps and the Nuremberg Code (Freyhofer 2004) that paved the way for the Declaration of Helsinki (World Medical Association 2008). It was codified and operationalised through the Belmont Report (National Commission for the Protection of Human Subjects of Biomedical and Behavioral Research 1979), which established the principles of biomedical ethics that remain dominant in the field (Beauchamp and Childress 2009): autonomy, justice, beneficence and non-maleficence.

Biomedical ethics has been hugely influential and underpins discussion of the human rights of patients (Council of Europe 1997). One crucial aspect of biomedical ethics is that it has been implemented via the well-established process of research ethics, based on ethics review, conducted by institutional review boards or research ethics committees, overseen by regional or national boards and strongly sanctioned by research funders, publishers and others.

There can be little doubt that this institutional strength of biomedical (research) ethics is a central factor guiding the AI ethics debate and leading to a principle-based approach that can be observed in most guidelines. This dominant position nevertheless has disadvantages. Biomedical ethics has been criticised from within the biomedical field as being overzealous and detrimental to research (Klitzman 2015). Empirical research on biomedical research ethics has shown inconsistency with regard to the application of principles (Stark 2011). And while largely uncontested in the biomedical domain, though not completely (Clouser and Gert 1990), the applicability of this approach to ethics in other domains, such as the social sciences, has been vehemently disputed (Schrag 2010).

There are two aspects from this discussion worth picking up for AI ethics. Firstly, there is the question of the implicit assumptions of biomedical ethics and their applicability to AI. Biomedical ethics was developed primarily to protect the rights of patients and research participants. This is no doubt transferable to AI, where the individuals on the receiving end of AI systems are worthy of protection. But because biomedical research predominantly aims to understand diseases with a view to finding cures, biomedical ethics is much less concerned with the purpose of the research. It is usually taken for granted that biomedical research pursues an ethically commendable goal: that of contributing to human health and thus to human wellbeing. Ethical concerns therefore do not arise from this goal itself but only from ways of achieving it. In the case of technical research, including AI research, it is not at all obvious that this implicit premise of biomedical research is applicable. The assumption that the research itself and its intended consequences are ethically acceptable and desirable is in need of much more questioning and debate, casting doubt on whether the process-oriented and principle-based biomedical research ethics process is a suitable one to base AI ethics on.

Secondly, biomedical principlism (Beauchamp and Childress 2009) leaves open the question of how to deal with conflicts between principles. This is a well-established problem for any ethical approach that is based on a set of non-hierarchical principles or values. In most cases it is possible to imagine situations where these come into conflict. Looking at the principles used in AI, it is, for example, easy to imagine a case where the principle of transparency would come into conflict with the principle of privacy. In order to successfully guide action or decision, the approach therefore needs to find ways of dealing with such conflicts. In addition, principlism has been criticised for being overly close to its US origins and not generalisable across the world (Schroeder et al. 2019).

Framing AI ethics in terms of human flourishing can address both concerns. By offering an overarching ethical ambition it proposes a point of comparison that can help address value conflicts. It also aligns more closely to 21st-century research ethics, which has been moving away from Western principles to global values (Schroeder et al. 2019). And it furthermore offers a perspective that does not take for granted that all research and technology innovation is desirable per se, but clearly posits flourishing as the overarching goal.
